# Comparison of Micro-Census Results for Magarya Ward, Wurno Local Government Area of Sokoto State, Nigeria, with Other Sources of Denominator Data

**DOI:** 10.3390/data4010020

**Published:** 2019-01-25

**Authors:** Margherita E. Ghiselli, Idongesit Nta Wilson, Brian Kaplan, Ndadilnasiya Endie Waziri, Adamu Sule, Halimatu Bolatito Ayanleke, Faruk Namalam, Shehu Ahmad Tambuwal, Nuruddeen Aliyu, Umar Kadi, Omotayo Bolu, Nyampa Barau, Mohammed Yahaya, Gideon Ugbenyo, Ugochukwu Osigwe, Clara Oguji, Nnamdi Usifoh, Vincent Seaman

**Affiliations:** 1 U.S. Centers for Disease Control and Prevention (CDC), Atlanta, GA 30333, USA; bkaplan@cdc.gov (B.K.); obb3@cdc.gov (O.B.); 2 National Stop Transmission of Polio (NSTOP), Asokoro, Abuja 900231, Nigeria; winidongesit@live.com (I.N.W.); ewaziri@afenet.net (N.E.W.); asule@afenet.net (A.S.); hayanleke@afenet.net (H.B.A.); naliyu@afenet.net (N.A.); umarkadi247@gmail.com (U.K.); dr.barau@gmail.com (N.B.); yahyakt@yahoo.com (M.Y.); ug.gideon@gmail.com (G.U.); uosigwe@afenet.net (U.O.); claraoguji@gmail.com (C.O.); maxiusifoh@gmail.com (N.U.); 3 Sokoto State Emergency Routine Immunization Coordination Center (SERICC), Sokoto Town, Sokoto State 840212, Nigeria; namalamf@gmail.com; 4 Sokoto State Primary Health Care Development Agency (SPHCDA), Sokoto Town, Sokoto State 840212, Nigeria; Shehuat66@gmail.com; 5 Bill and Melinda Gates Foundation, Seattle, WA 98109, USA; vincent.seaman@gatesfoundation.org

**Keywords:** immunization, Nigeria, target population, census

## Abstract

Routine immunization coverage in Nigeria is suboptimal. In the northwestern state of Sokoto, an independent population-based survey for 2016 found immunization coverage with the third dose of Pentavalent vaccine to be 3%, whereas administrative coverage in 2016 was reported to be 69%. One possibility driving this large discrepancy is that administrative coverage is calculated using an under-estimated target population. Official population projections from the 2006 Census are based on state-specific standard population growth rates. Immunization target population estimates from other sources have not been independently validated. We conducted a micro-census in Magarya ward, Wurno Local Government Area of Sokoto state to obtain an accurate count of the total population living in the ward, and to compare these results with other sources of denominator data. We developed a precise micro-plan using satellite imagery, and used the navigation tool EpiSample v1 in the field to guide teams to each building, without duplications or omissions. The particular characteristics of the selected ward underscore the importance of using standardized shape files to draw precise boundaries for enumeration micro-plans. While the use of this methodology did not resolve the discrepancy between independent and administrative vaccination coverage rates, a simplified application can better define the target population for routine immunization services and estimate the number of children still unprotected from vaccine-preventable diseases.

## 1. Introduction

Routine immunization (RI) coverage in Nigeria is suboptimal, with independent sources reporting a national infant coverage rate for the third dose of Pentavalent vaccine (Penta3) at 38% in 2013 (Nigeria Demographic and Health Survey (DHS)) [1] and at 33% in 2016 (Multiple Indicator Cluster Survey (MICS)) [2]. These surveys were considered for the World Health Organization/UNICEF Estimated National Immunization Coverage (WUENIC), which reported a Penta3 coverage of 42% for 2017 [3]. The states of Nigeria report high variability in coverage, with overall lower coverage in the northern states. For Sokoto state, in the northwestern part of the country, the 2016–2017 MICS reported Penta3 coverage to be 3% [2]. However, for the same state and year, the administrative coverage reported for Penta3 (calculated by dividing the number of doses administered to infants by the estimated target population) was 69% [4]. It is unclear what drives the discrepancy between administrative and independent survey coverage estimates. Possibilities include inflated numerators, deflated denominators, or a combination of both biases.

Accurate population data are an essential component of reliable estimates for RI coverage, as they give public health officials a baseline to assess the performance of the national Expanded Programme on Immunization (EPI). However, multiple stakeholders report concerns regarding the official population projections from the 2006 Nigerian Population and Housing Census [5], which is used to calculate the administrative vaccination coverage rate for RI antigens based on state-specific standard population growth rates. Operations for supplementary immunization and surveillance activities use population estimates from other sources (such as satellite imagery/geographic information system (GIS) information [6] and house-to-house enumeration for supplementary immunization activities (SIAs)) [Gidado, S. NSTOP, Abuja, Nigeria, personal communication, 15 May 2018], but there have been no efforts to date to conduct an independent validation of these approaches. The Government of Nigeria Federal Ministry of Health (FMoH) and the National Primary Health Care Development Agency (NPHCDA) are aware of these concerns and support efforts to develop more reliable population estimates.

**Figure 1.**
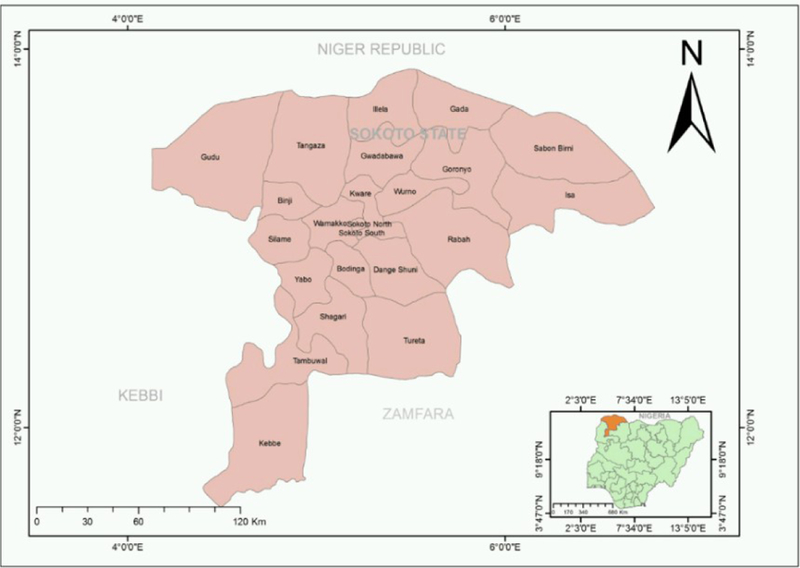
Map of Sokoto state and its local government areas (LGA).

**Figure 2.**
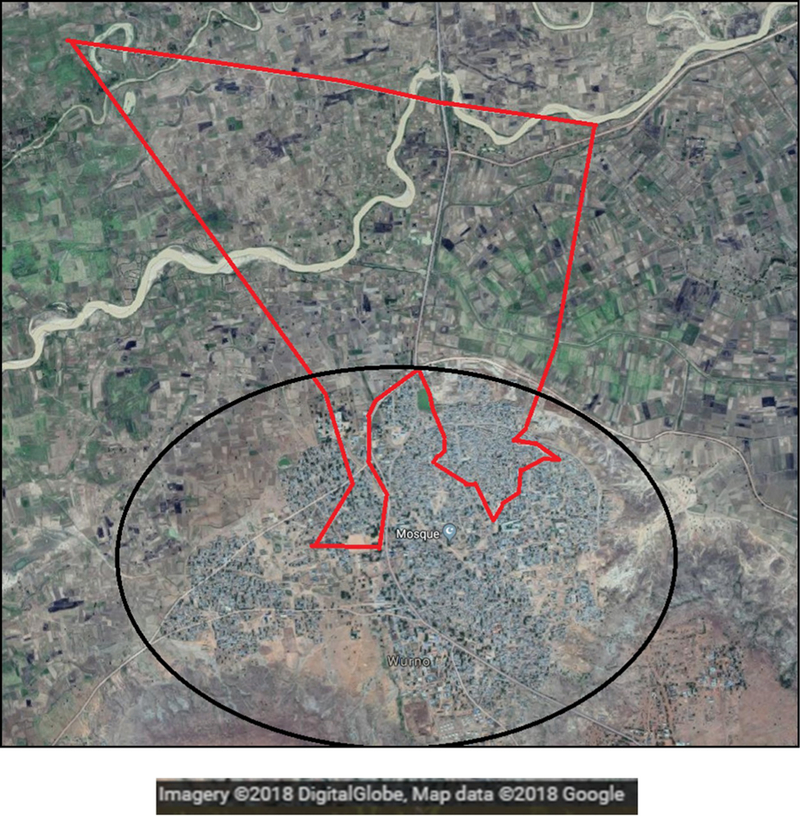
Satellite image of Wurno town (black circle) and Magarya ward (red boundaries). Source: GoogleMaps (accessed on 1 November 2018).

We conducted a micro-census in Magarya ward, Wurno local government area (LGA) of Sokoto state, Nigeria, during July 11–17, 2018 (Figure 1). Our goal was to obtain an accurate count of the total population present (residing and visiting) in the ward, and compare these results with those from other sources of denominator data, stratifying by age and settlement. We developed a precise micro-plan using satellite imagery, and supported operations in the field using a navigation tool to guide teams to each building assigned to them, without duplications or omissions. The particular characteristics of the selected ward—with a large uninhabited area in the North, and two densely populated prongs in the south that include only portions of Wurno town (Figure 2)—underscored the importance of using a precise shape file and micro-plan to follow pre-established boundaries and prevent teams from crossing into neighboring wards.

## 2. Results

### 2.1. Demographic Analysis

#### 2.1.1. Settlement Characteristics

The micro-census collected information for 8601 persons found in Magarya ward during the six days of the enumeration. These persons were located in 26 settlements, 2488 buildings (of which 1576 were residential and inhabited), 2632 dwellings/flats (of which 1514 were occupied), and 1704 households. See Section 3.5 for a description of the enumeration units.

#### 2.1.2. Household Characteristics

The overall average number of household members was 7.2 (SD = 3.9) across all settlements, ranging between 3.6 to 9.2 by settlement. Among the 1704 households, 1378 (80.9%) described the household head as monogamous, and 237 (13.9%) as polygamous. However, when we counted the number of spouses per household head, we found that 395 household heads (25%) reported two or more wives. Also, 16 households (1%) were composed of visitors (i.e., families who were not permanent residents of the ward, but were staying with relatives in the same dwelling/flat on the day of the enumeration), eight households (0.5%) were composed of residents of collective quarters (i.e., persons who were not related by blood or marriage, but established communal living arrangements—for example, pupils at a boarding school, inpatients at the hospital), and four households (0.2%) were composed of nomadic persons (i.e., families who define themselves as nomadic and were interviewed in Magarya ward on the day of the enumeration). These household categories were mutually exclusive.

#### 2.1.3. Personal Characteristics

##### Gender:

**Figure 3.**
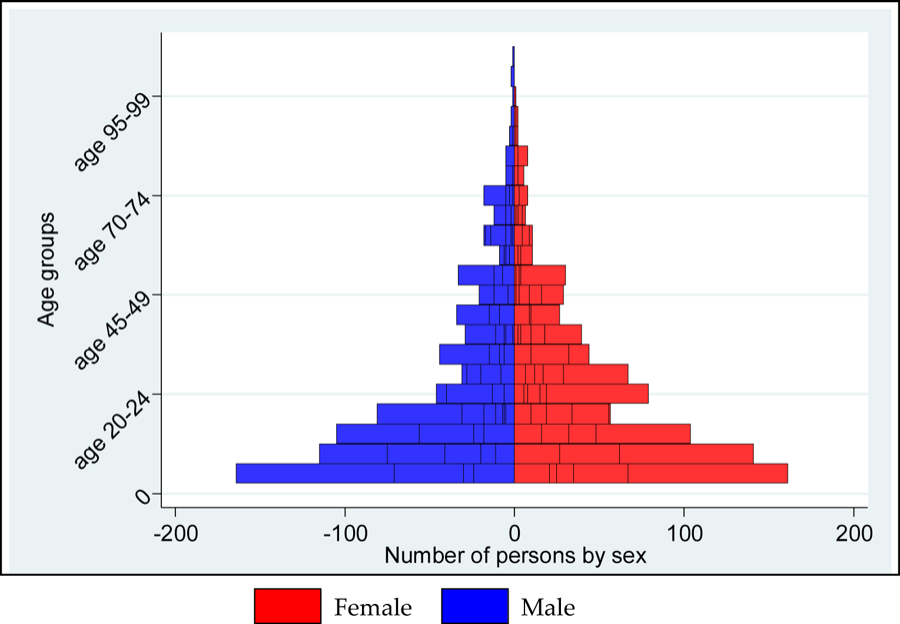
Population distribution by age group and sex, overall.

**Figure 4.**
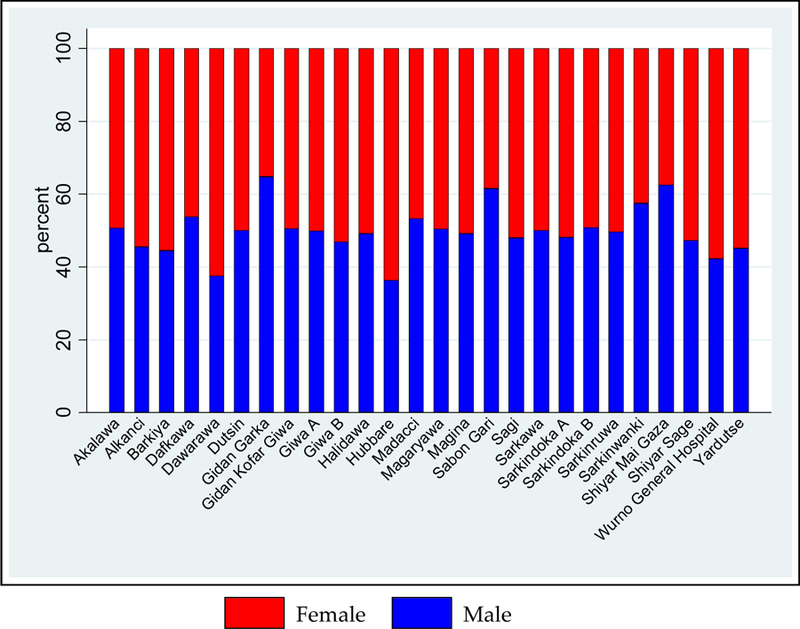
Population distribution by sex and settlement.

The number of males and females was 4282 (49.8%) and 4314 (50.2%), respectively (Figure 3). An equal proportion of males and females was present in all but 4 of the 26 settlements (Figure 4). Among children younger than five years of age, the proportion of males and females was similar. However, in the 20–40 year age group, the proportion of the total population who were women was 34% and who were men was 26% (Figure 4).

##### Age:

The average age household member was 20.4 years (range: 0–105), and was similar among men (20.9 years) and women (20.0 years). There was age heaping around “round” numbers (e.g., 30, 35). Among the 1581 household heads, the average age was 45.7 years (range: 13–105). Among their wives, the average age was 31.0 (range: 13–80). There were 240 (2.8%) children younger than 12 months, 1599 (18.6%) children younger than five years, and 4271 (49.7%) children younger than 15 years. Age groups were not mutually exclusive to facilitate comparison with other sources of denominator data. Age group data by settlement are indicated in Table 1.

##### Relationship to household head:

Overall, 1341 (84.9%) household heads were reported as male and 238 (15.1%) were reported as female. On average, each household head had 3.0 children (range: 0–19). This average value varies considerably among settlements, from Gidan Garka (1.0 children) to Shiyar Mai Gaza (5.6 children).

### 2.2. Comparison with Other Sources of Demographic Data

#### 2.2.1. Comparison with the 2018 projections from the 2006 Population and Housing Census [5]

The population projections for 2018 were derived from the 2006 Census, where a Sokoto-specific 3.0% constant growth factor is applied each year to the demographic totals collected in 2006. Children younger than 12 months are estimated to be 4.0% of the total population of Sokoto state. These projections are available only at the ward level.

As the population projections for 2018 are available only at the ward level, we could not compare population size by settlement. Nonetheless, a comparison of the totals by age group revealed interesting differences. The micro-census counted 240 children younger than 12 months (2.8% of the entire population), while the 2018 projections estimated 680 children in the same age group (4.0% of the population). For children younger than five years of age, the census projections estimated 3390 children (20.0%) compared with the micro-census’s count of 1599 (18.6%). For the age group of children younger than 15 years of age, the census projections estimated 8089 children (47.6%) compared with the micro-census’s count of 4271 (49.7%). The difference between these age-specific proportions decreased as age increased. The absolute count of the micro-census remained approximately half of that projected by the census. Finally, the total ward size was 16,994 based on the Census projections, and 8601 based on the micro-census.

#### 2.2.2. Comparison with the 2017 Polio Emergency Operations Centre (EOC) Walk-Through Enumeration

House-to-house walk-through enumeration of children younger than 15 years of age in all settlements selected for polio SIAs was conducted in June 2017 by the National Polio EOC. Its purpose was to strengthen the micro-plan for the national polio vaccination campaign in Sokoto state

The house-to-house walk-through enumeration completed by the Polio EOC in June 2017 reported results by settlement. This aggregation allows us to compare the size of each age group (up to 15 years) on a more granular level. Not all the settlements listed during the micro-census appeared in the walk-through results. This occurs because the micro-census enumerators listed the names of settlements as reported by the respondents, while the walk-through micro-plan used the official settlement names. In 12 of the 18 settlements with matching names in the two databases, children younger than 12 months represented a smaller proportion in the EOC walk-through results compared with the micro-census results (Table 2). The same comparison holds true for children younger than five years in all settlements (Table 3). The proportion of children younger than 15 years was assumed to be 20.0% of the total population in all settlements (Table 4). From there, the total population estimate was calculated to be 26,205—the highest among the different data sources (Table 5).

#### 2.2.3. Comparison with the Count of Children Younger than 12 Months for 2018 Demand Generation Project (Franka, R.; Abad, N. CDC, Atlanta, GA, USA, personal communication, 15 May 2018)

This refers to the monthly count of all children younger than 12 months, conducted by local leaders. This project was launched in March 2018 in nine LGAs of Sokoto—including Wurno LGA, where Magarya ward is located. The goal is to update the target population for each health facility offering RI services, and identify children lost to follow-up

The August count of newborns conducted by settlement heads was compared to the number of children younger than 12 months identified by the micro-census. Given the parameters of the project, we could only compare the absolute reported number of children younger than 12 months with the result obtained during the micro-census, with no reference to their proportion in the population at large (see Table 2). During the August count, the settlement heads recorded 238 children younger than 12 months in Magarya ward, which differs from the findings of the micro-census by only two children.

#### 2.2.4. Comparison with the 2018 Geographic, Population, and Demographic Estimates (GeoPoDe) [6]

The Bill and Melinda Gates Foundation (BMGF) calculated population totals for different age groups down to the settlement level. It used nationally representative household surveys and their cluster locations to predict the proportion of the under-five population age group in a 1 × 1 km area using a Bayesian hierarchical spatio-temporal model [7]. The under-one age group in Magarya ward was estimated using a fixed 3.8% proportion of the total population, which was derived from modeled data from the 2008 DHS.

These population totals are available online at the Geographic, Population, and Demographic (GeoPoDe) website. For Magarya ward, the reported total population was 5,439 [8]. GeoPoDe’s ward boundaries also were obtained from DigitalGlobe [9] through GoogleMaps, and thus exactly match those used by the U.S. Centers for Disease Control and Prevention’s (CDC) Geospatial Research, Analysis, and Services Program (GRASP). However, because we were forced to slightly expand our operational boundaries to follow roads and other natural demarcations, BMGF staff re-calculated the population size by sex and age group using the exact same boundaries as the micro-census (“revised GeoPoDe boundaries”). This was done using the geo-coordinates collected in the field for each building.

Using revised GeoPoDe boundaries, the total population size was estimated to be 8,708. As in other comparisons, children younger than 12 months represented a larger proportion of the total population in the GeoPoDe results compared with the micro-census results. The same comparison holds true for children younger than 5 years and younger than 15 years.

#### 2.2.5. Comparison with the 2013 Nigeria Demographic and Health Survey [1]

**Figure 5.**
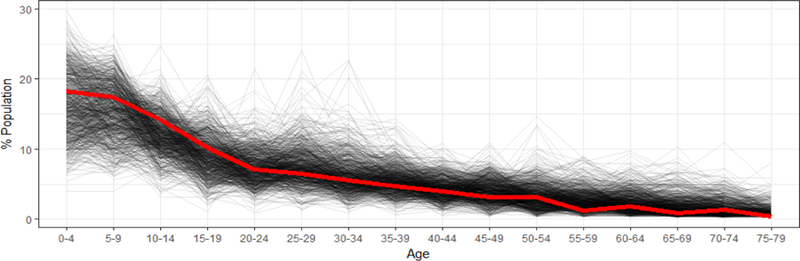
Age distribution of population reported in the Magarya micro-census and the national 2013 DHS. (Courtesy of Christopher Jochem, Department of Geography & Environment, University of Southampton, 2018).

The 2013 Nigeria Demographic and Health Survey (DHS) is a national sample survey that provides information on background characteristics of the respondents. The sample for the 2013 DHS was nationally representative and covered the entire population. In Sokoto, 1,080 completed interviews were conducted [1]. We selected the 2013 DHS because it is an independent survey of the Nigerian population, and is often used for secondary analyses and validation purposes [10–12]. The DHS dataset available to the public aggregates data to the state level only, so a direct comparison with the Magarya micro-census is not possible. Nonetheless, some high-level assessments are still relevant. In the micro-census, the proportion of males to females was 49.8% to 50.2% (Figure 3), and in the DHS, the proportion was 49.5% to 50.5% [1]. The average number of children per household head was 3.3 in the 2013 DHS, and 3.0 in the micro-census. In Figure 5, the age distribution reported in the micro-census (heavy red line) is consistent with the age distribution estimated for the DHS clusters (individual black lines). According to the 2013 DHS, the average age in Sokoto is 19.8 years (range: 0–90), while it is 20.4 years in the micro-census. Age heaping is an issue in the DHS as well. In Table 4, children younger than 12 months constituted approximately 4.2% of the state’s population based on DHS results. This proportion is higher than that reported from the micro-census count for Magarya ward alone (2.8%)

## 3. Materials and Methods

### 3.1. Ethical Considerations

This micro-census is part of the Demand Generation project, which was designed by the U.S. Centers for Disease Control and Prevention (CDC) to strengthen RI coverage in Sokoto. This project is currently underway in nine LGAs of Sokoto state, and includes monthly listing of all children younger than 12 months (including name, age, and settlement of residence) by the settlement head, reconciliation with healthcare facilities, follow-up on vaccination defaulters, and community mobilization to increase and sustain attendance during RI sessions. The micro-census took place in a single ward using more rigorous strategies and tools to count children younger than 12 months than in the overall project. The micro-census received approval from the Health Research Ethics Committee in Sokoto state and was determined to be non-research by the Human Research Protection Office of the U.S. CDC Center for Global Health. The Sokoto State Emergency Routine Immunization Coordination Center (SERICC) and the state’s Emergency Operations Center (EOC) granted approval for the implementation of this micro-census exercise in May 2018. The data collection and analysis process were carried out following the rules of the Declaration of Helsinki of 1975, revised in 2008 (https://www.wma.net/wp-content/uploads/2018/07/DoH-Oct2008.pdf).

### 3.2. Target Population

Because the target population for RI services is comprised of children younger than 12 months, the micro-census could have included only this subset of the population. However, a simple count of these children does not provide information on the overall age distribution, which is often pre-set despite variations observed between and within states and LGAs. This micro-census was a de facto count of persons of all ages found in the ward on the day of the census. We chose this enumeration option based on two considerations. First, although the target population for RI services is the estimated number of newborns per year among residents, health facilities are required to immunize all children who attend RI vaccination sessions, regardless of residence. Second, the other sources of operational population estimates used de facto enumeration as well [5,6].

### 3.3. Location

The micro-census exercise took place in Magarya ward, in Wurno local government area (LGA) of Sokoto state. This state was selected for two reasons. First, Sokoto reports one of the largest discrepancies in Nigeria between administrative and independent survey vaccination coverage rates [2,4]. Therefore, the state urgently requires additional verification of its population size to identify missed children and develop strategies to vaccinate them. Second, because of low survey vaccination coverage, the Demand Generation project is being conducted in Sokoto. Wurno LGA was selected because it reported no security concerns at the time of the micro-census, and because it is close to Sokoto Town, where the enumeration teams resided during this exercise. Magarya ward was selected based on its small total population (<9000 people), which allowed enumerators to implement a full census in a short period of time. The ward did not host large markets or gatherings during the days of the micro-census. Magarya ward includes 16 settlements and a total population of 8708 persons based on standardized assumptions about the number of residents in structures identified by satellite imagery (Table 3) [8].

### 3.4. Mapping

On 5 April 2018, GRASP downloaded high-resolution satellite imagery for Magarya ward from DigitalGlobe, a platform for geospatial information [9]. We uploaded the image for the ward into ArcGIS® (version 10.5, Esri, Redlands, CA, USA) [13] and recorded the latitude and longitude of each visible building that fell within the reported boundaries of the ward. These coordinates were aggregated into small enumeration areas (EAs), which included 20–36 buildings each and constituted the workload for one enumeration team for one day (Appendix A). During this design phase, we expanded the boundaries of the ward slightly so the EAs would follow natural demarcations in the field (e.g., roads, compound walls). Because our EAs covered the entire ward, no dwelling was missed. Satellite imagery was successfully used to prepare detailed micro-plans for polio vaccination campaigns in Nigeria, countries in the Lake Chad basin (Cameroon, Chad, Niger), Somalia, and remote areas of the Democratic Republic of the Congo (Kaplan, B. CDC, Atlanta, GA, USA., personal communication, 12 March 2018).

We converted the satellite image of each EA into a geoPDF file (i.e., a PDF file with embedded geo-coordinates), and uploaded them into the open-source Android application EpiSample® version 1 [14] to generate a list of buildings. To cover the entire ward in seven days, we divided it into 129 EAs and distributed them among 21 enumeration teams. The application used global positioning system coordinates and a compass to guide the enumeration team to each building. Enumerators relied on local staff to identify previously unrecorded buildings (e.g., new constructions, nomadic camps).

### 3.5. Enumeration Units

#### 3.5.1. Building

A building is any independent free-standing structure (e.g., stand-alone house, structure within a compound, apartment complex, makeshift structure) comprising one or more dwellings, flats, or other spaces, covered by a roof and usually enclosed within external walls or dividing walls that extend from the foundations to the roof. However, in tropical areas, a building may consist of a roof with supports only, that is, one without constructed walls [5]. In some cases, a roofless structure consisting of a space enclosed by walls may be considered a building. Structures that are not intended for habitation (ex., garages, barns, classrooms), but are occupied as living quarters by one or more households at the time of the micro-census, are treated as inhabited structures. Among nomadic and refugee/internally displaced people (IDP), a tent or makeshift edifice is considered a structure. For this enumeration, we could not use the satellite image to determine whether a building was inhabited. Therefore, the teams visited each building in the ward and conducted interviews only in residential structures.

#### 3.5.2. Dwelling

The dwelling (or flat) is a housing unit or accommodation occupied by one or more households with a single main entrance that leads directly outside in the open or into a public corridor or hallway [5]. For face-to-face, or room-by-room type of building, each room or set of rooms occupied under one arrangement with one recognized tenant is a housing unit.

#### 3.5.3. Household

A household is comprised of a group of persons living together under the same roof or in the same building/compound, who eat from the same pot and recognize themselves as a unit [5]. This definition stresses the importance of the nuclear family as the unit of enumeration, and allows enumerators to divide extended families and dwellings into manageable units. At times, members may belong to special types of populations, and their households require specific enumeration strategies.

##### Polygamous households:

According to the 2010 Nigeria General Household Survey [5], 31.3% of married persons in Sokoto are in a polygamous marriage. For the purposes of enumeration, we collected information from the male household head for all his wives and children. If the head was female, we collected information on family members directly from her.

##### Nomadic households:

The exact size of Sokoto’s nomadic population is unknown, but it likely comprises a substantial percentage of the total population given this state’s position along well-known nomadic routes in the Sahel. In the absence of permanent dwellings, the enumerators identified any nomadic groups staying in the ward at the time of the micro-census, and counted the households based on the standard definition [12].

##### Refugee/IDP groups:

We define IDPs as persons who left their original location of residence within Nigeria and moved into Magarya ward. IDPs reside in one of two locations: (a) in the community, as guests of friends or relatives. In this case, the enumerator counts these persons along with the more permanent residents; or (b) in special camps/neighborhoods where they are the only occupants. In this case, the enumeration team visits the camp and counts the households based on the standard definition. The same instructions apply to refugee populations residing in the ward [15].

##### Visitors:

Like with IDPs and refugees, the enumerator counts persons who are visiting the selected ward on the day of the census. Depending on the living arrangements in place, these persons are included in the host’s household or form a separate unit.

##### Collective quarters:

These include hospitals, barracks, prisons, orphanages, and so on. The distinguishing feature is that these households do not have a head. For the purposes of enumeration, the room where the persons sleep is counted as the dwelling, and all persons assigned to that room constitute a household [15]. The enumerator assigned the role of household head to the oldest person.

#### 3.5.4. Household Member

A person who is currently staying in Magarya ward, regardless of sex or age. A household member normally lives together with other household members in one house or closely related premises, and takes his/her meals from the same kitchen. She/he may or may not be related to the other household members by blood or marriage, including a house-helper or farm-laborer.

### 3.6. Data Collection Tool

Data were collected using an Open Data Kit (ODK) [16] data collection form designed specifically for this micro-census. The form automatically assigned a unique identification number to each building, and each dwelling, household, and household member was subsequently nested within its building of residence. This nesting structure was reconstructed during the analysis process to exactly locate each person within his/her place of residence.

For each enumeration level, the ODK form collected the identification number and type of the unit. For each person, the ODK form collected name, sex, age, date of birth, and relationship to household head. For those who did not know their exact age, approximate age was recorded. If the building was not residential and not inhabited (e.g., school, shop, clinic without inhabitants), the team recorded it as “non-residential” and proceeded to the next structure.

### 3.7. Enumeration Procedures

The engagement of community leaders was generated by local-based staff of the National Stop Transmission of Polio (NSTOP) program, which was established in 2012 to create a network of staff working at national, state, and district levels in areas deemed high risk for vaccine-preventable disease outbreaks [17]. Town announcers promoted the micro-census across the ward during the two days preceding the activity and throughout the enumeration period, 11–17 July, 2018.

The enumeration team was composed of one navigator, one enumerator and one local field guide. The involvement of field guides was key in obtaining the cooperation of responders. Seven supervisors—residents of the Nigeria Field Epidemiology and Laboratory Training Program (FELTP) [18]—participated in this activity, with one supervisor responsible for three teams. Each building was associated with latitude and longitude values. The team visited each building located in their daily EA. If a building was not pre-recorded, the team captured its coordinates and added it to the list. The ODK software launched a blank questionnaire for each building. If the building was residential, the team collected data on each dwelling, household, and household member. Each building was associated with latitude and longitude values.

Once inside the building, the team counted the number of dwellings (i.e., housing units or flats) and households (i.e., family units). Verbal consent from household heads or their proxy to participate in the micro-census was obtained, and each person had the option to refuse participation. Only three households declined to participate in the micro-census, and did not provide a reason for their refusal. Household members could also provide information on family members who were not present at the time of enumeration, but would return that evening (e.g., farmers, shopkeepers, students).

All interviews were conducted in the local version of the Hausa language. The database was transmitted to the NSTOP server in Abuja at the end of each day, and collated into a single dataset.

### 3.8. Data Quality Checks

The protocol for this micro-census exercise incorporated numerous validity checks at each step of the planning and implementation process. This was done to counteract some of the most common data quality issues encountered in other censuses. For example, the boundaries reported in the shape files of wards and LGAs do not always match the demarcations set in the field (Seaman, V., Bill and Melinda Gates Foundation, Seattle, WA, USA, personal communication, 17 February 2018). Therefore, teams may overstep the boundaries of their area of work, cover a smaller portion than assigned, or visit the same areas twice. Also, some data points are more difficult to elicit correctly. For example, imprecise information about a person’s date of birth is a limitation for many enumeration exercises [19–22]. Even if all data points are elicited correctly, their transcription in data collection forms (paper or electronic) may generate data entry errors that cannot be rectified during analysis [23]. In the field, limited supervision facilitates these mistakes in data collection and entry, because data are not objectively reviewed before submission [24]. Also, poor monitoring makes the consistent application of procedures more difficult across teams [25]. Finally, delays in data review and analysis hide systematic data collection errors that could otherwise be rectified in the field [25]. The sections below describe the data validation steps we took to minimize these issues.

#### 3.8.1. Pre-Implementation Phase

The use of satellite imagery and the EpiSample navigational tool eliminated the possibility of missing some areas or buildings in Magarya ward. These same tools ensured that the EAs did not overlap, minimizing the possibility of duplication. This categorization was further validated by an independent reviewer, who repeated this digitization work to finalize the list of buildings. This precise micro plan allowed us to determine the exact number of days required for this exercise. Also, the design of the ODK data collection tool minimized the possibility of entering duplicate information for dwellings, households, and household members, and included pre-programmed skip patterns that minimized data entry errors during collection. For example, if a household reportedly included five members, the ODK form repeated the person-level battery of questions five times before moving to the next household, so that information on each member was captured without fail. Also, the age variable was restricted between 0 and 118 years, and age of birth could not be later than the date of the micro-census.

#### 3.8.2. Implementation Phase

The low supervisor-to-team ratio (3:1) ensured that supervisors supported each member of the team and closely monitored the fieldwork, data entry procedures, and data submission. Our micro plan ensured that the three teams under the same supervisor were assigned to EAs close to one another, so the supervisor did not have to walk great distances. Also, as a check on duplicate counting, we collected the name of each person within the household and added an item in the questionnaire asking if the person had been interviewed before for this micro-census. Only 77 persons answered affirmatively, and their duplicate records were dropped from the dataset.

#### 3.8.3. Post-Implementation Phase

We conducted validity checks on the incoming data each evening to identify any data collection problems and provide detailed real-time feedback to the teams. These pre-established validity checks identified missing variables and corrected data entry errors where possible. For example, we used date of birth information to correct or complete the age variable. We verified that no child was listed as older than his/her parent, or that all sons were classified as “male”. We also verified that only residential dwellings included households. Finally, we plotted the age and sex distribution for each settlement, to assess whether males and females were equally represented in each settlement. Sex information was missing for 42 (0.5%) persons. When we encountered any questionable data entries or patterns, we informed the field teams to obtain explanations and provide feedback, so the issue would be correct in the next day’s data.

### 3.9. Data Analysis

We used Stata 13 for all data management and analysis procedures. We collated all data points into a single database, and we ran descriptive statistics to identify missing values and outliers for each variable. First, we tabulated the number of settlements, dwellings, households, and persons interviewed each day, and verified that the nesting structure was in place (e.g., no households residing in unoccupied dwellings, or five persons assigned to a household of three members). Second, we tabulated each variable to identify whether more than 10% of observations was missing. Third, we cross-referenced each variable by settlement, to assess whether missing information was more prevalent in some teams compared with others. Fourth, we cross-referenced different combinations of individual-level variables to identify inconsistent data entries and, if possible, correct them. These validation checks were described in Section 3.8.3.

Each variable was stratified by settlement, age, and sex to examine demographic distributions. We compared the results of the micro-census with those from other operational denominator sources. We examined the size and direction of the discrepancies, as well as the methodological differences that may have led to these.

## 4. Discussion

### 4.1. Summary of Findings

The enumeration methods described in this report are used to conduct national censuses around the world, including the Nigeria 2006 Population and Housing Census. Previous studies used mapping infrastructure (such as Digital Globe [9]) to develop public health maps for advocacy, training, and emergency response [26,27]. Others combine geolocated cluster survey data with geospatial layers to predict target population proportions and coverage of public health interventions [28,29]. The novel aspect of this activity was the combination of satellite imagery, geocoding methods, and navigational tools to develop a precise micro plan and guide the enumeration teams in the field.

The first objective of this micro-census was to obtain an accurate count of the total population living in Magarya ward, in Sokoto state. We counted 8601 persons in the ward. Of these, 240 (2.8%) are children younger than 12 months and 1599 (18.6%) are younger than five years. The age heaping we noticed around “round” numbers might be related to the uncertainty of precise age by many in the population [30]. The sex distribution was roughly equal among men and women at all ages. The only exception was observed for men in the 20–40 year age group, who seemed to be under-represented. This absence might be consistent with labor migration outside the ward. As this is a de facto enumeration, persons not present on the day of the micro-census were not counted.

In each household, the average number of members was 7.2. The average number of children per household head was 3.0. Among the 1704 households, 1378 (80.9%) household heads describe themselves as monogamous and 237 (13.9%) as polygamous. However, approximately 25% of household heads reported having two or more wives, suggesting that there may have been some errors in the classification of household type. About 15% of household heads were female. This categorization is likely because of the absence of the male household head at the time of the enumeration, rather than the recognition of a woman as head of the household.

Finally, the distribution of households across buildings and dwellings/flats suggests the following: a) almost half of the buildings in the ward were uninhabited; b) inhabited buildings contained multiple dwellings/flats, some of which were not occupied; and c) many of the dwellings/flats might have been occupied by more than one household.

### 4.2. Comparisons

The second objective was to compare the results of the micro-census with those from other sources of denominator data. In all comparisons, we found our number and proportion of children younger than 12 months and younger than five years to be smaller compared with other estimations. Multiple interpretations explain this discrepancy. The micro-census might have counted fewer persons in each age group compared with other enumeration exercises because we strived to avoid duplicate counts. Also, the uncertainty around the reported age—especially among younger children—might have contributed to the creation of imprecise age groups that make comparison between data sources difficult. It is possible that some children younger than 12 months were reported as being older (Pullum, T. The Demographic and Health Surveys Program, ICF, Rockville, MD, USA. Personal communication, 2018). However, as more people were added to each age group, the discrepancies seemed to smoothen. Another possibility is that variations in age trends within and across wards might be greater than those estimated by the GeoPoDe exercise. The use of assumed fixed proportions to estimate age group size does not reflect the reality in the field, and inflates the count of infants in some settlements.

When interpreting our findings, we must consider the shape of Magarya ward. Its boundaries are irregular, and the southern portion is enmeshed with Marafa ward, where the LGA capital Wurno is located (see Figure 2). There are no physical barriers between the two wards (e.g., river, main road), so enumeration teams may easily overstep the established boundaries if the micro plan is not exact. This may lead to inflated results for other enumeration methods. This interpretation gains strength when we compare our results to those revised from GeoPoDe. These estimates were calculated using the exact same boundaries as the micro-census, using the geo-coordinates of each building visited. As shown in Table 3, our total population count is only 107 persons fewer (1% of the micro-census population). This comparison underlines the need for standardized boundary shape files, so each enumeration exercise observes the exact same area and results are analogous. Our precise micro plan also minimized the chance of duplicate or erroneous counts. Because no other enumeration methodology achieved this level of precision, we would expect our count to be lower.

### 4.3. Strengths and Limitations

#### Planning:

This micro-census required five months of planning (February through June 2018) and seven days for implementation (11–17 July, 2018). This long planning period allowed us to include key best practices that strengthen our implementation operations in the field.

#### Involvement of state and local authorities:

The early engagement of officials from the Sokoto SERICC, the Sokoto EOC, and the LGA- and ward-level authorities ensured their approval and support when conducting trainings, field preparations, and the enumeration itself.

#### Involvement of community leaders:

The collaboration of community leaders ensured that residents could be informed of the proceedings of the exercise and participate freely.

#### Satellite images:

We used recent satellite images of Magarya ward, where each building was clearly visible. These images are already available to the Nigerian National EOC.

While the execution of the activity was successful, we faced multiple limitations during planning and execution. Some limitations could not be partially or fully addressed.

#### Single ward enumeration:

This exercise included a single ward in Sokoto state because organizers wanted to assess the validity of this methodology before investing time and resources in larger areas. This dataset does not allow for any extrapolation to a larger area (e.g., LGA, state), as the activity was not designed to perform this task. The results of this micro-census are pertinent to Magarya ward alone, but they do suggest issues that may be relevant to other enumeration exercises.

#### Imprecise information on age:

On the one hand, we collected information on persons of all ages, so no one was excluded based on erroneous age information. On the other, the estimated age groups may not be precise. This problem remains a concern when trying to estimate the number of children eligible for RI services.

#### Age displacement:

Younger children may systematically be reported as older than they actually are. This phenomenon has been observed before in other enumeration exercises in developing countries [21,22,31]. Therefore, some of the children reported in older age groups may actually belong to the youngest group. Without precise information on date of birth, it is impossible to reconstruct the correct groupings.

#### Data collection in the household:

A local field guide accompanied each enumeration team to facilitate interactions with household members. All enumerators were women, allowing easy access to dwellings and families. Nonetheless, the enumeration teams may have missed persons temporarily absent from the house or the ward. Also, obtaining information from women when the household head was not present was challenging at times, especially when the information concerned the husband. This is a restriction in Sokoto communities, and might have affected the enumeration of men aged 20–40 if they were outside the house at the time of the micro-census, but residing in the ward.

## 5. Conclusions

This micro-census methodology allows enumerators to control their area of operation with surgical precision, thus avoiding any duplications or overlap with other wards. In addition, the navigational tool ensured that enumeration teams reached every building in the ward, and could add buildings to the EA list where needed. The peculiar shape of Magarya ward further emphasizes these advantages, because we were able to follow the established boundaries exactly, while other enumeration methodologies may not have been as precise.

Future enumeration exercises in Nigeria can take advantage of this technique to better define the target population for RI services in a given area. Depending on time and resources available, this methodology can be expanded to include larger areas wherever satellite images are available. As in this example, it may be used to validate the enumeration results from other exercises and assess the size and direction of any discrepancy. Most importantly, it offers an opportunity to better understand how many children require RI services in Nigeria and, therefore, how many must still be reached to ensure high coverage against vaccine-preventable diseases. Although other enumerations may be over-counting the RI target population, the degree of denominator discrepancies found in our comparisons may not account for the marked differences in survey versus administrative coverage levels in Sokoto.

## Figures and Tables

**Table 1.** Proportion of children younger than 1 year, 5 years, and 15 years, by settlement.

Settlement.	Total Population	Under 1 Year		Under 5 Years		Under 15 Years	
		N	%	N	%	N	%
Unknown	142	1	0.7	25	1.6	66	1.6
Akalawa	994	28	1.8	183	11.4	487	11.4
Alkanci	143	3	2.1	33	2.1	77	1.8
Barkiya	135	4	3.0	19	1.2	64	1.5
Dafkawa	223	9	3.6	48	3.0	108	2.5
Dawarawa	72	4	5.6	15	1.0	35	0.8
Dutsin	226	5	2.2	52	3.3	113	2.7
Gida Garka	17	0	0.0	4	0.3	6	0.1
Gida Kofar Giwa	95	2	2.1	18	1.1	50	1.2
Giwa A	571	21	2.5	96	6.0	284	6.7
Giwa B	130	3	0.0	17	1.1	59	1.4
Halidawa	238	8	0.8	47	2.9	119	2.8
Hubbare	22	1	4.6	5	0.3	8	0.2
Madacci	60	1	1.7	7	0.4	23	0.5
Magaryawa	1,271	24	1.3	203	12.7	602	14.1
Magina	214	6	2.8	43	2.7	108	2.5
Sabo Gari	26	0	0.0	7	0.4	13	0.3
Sagi	146	3	1.4	19	1.2	80	1.9
Sarkawa	538	20	3.2	107	6.7	265	6.2
Sarkindoka A	1,563	52	2.3	326	20.4	791	18.5
Sarkindoka B	705	15	2.1	138	8.6	379	8.9
Sarkinruwa	363	12	3.3	65	4.1	188	4.4
Sarkinwanki	120	2	0.8	14	0.9	50	1.2
Shiya Mai Gaza	48	1	2.1	10	0.6	21	0.5
Shiya Sage	246	9	3.7	48	3.0	128	3.0
Wurno General Hospital	71	0	0.0	5	0.3	30	0.7
Yardutse	222	6	2.3	45	2.8	117	2.7
**Total**	**8601**	**240**	**2.8**	**1599**	**18.6**	**4271**	**49.7**

**Table 2.** Under-1 year population estimates, by settlement in the Magarya micro-census and Emergency Operations Center (EOC) house-to-house count.

Settlements	Micro-Census Count		EOC House-to-House Count		Demand Generation Count	

	N	%	N	%	N	%
Unknown	1	0.7				
Akalawa	28	1.8	84	2.6		
Alkanci	3	2.1				
Barkiya	4	3.0	24	1.5		
Dafkawa	9	3.6	19	0.9		
Dawarawa	4	5.6	18	2.1		
Dutsin	5	2.2	19	2.6		
Gida Garka	0	0.0				
Gida Kofar Giwa	2	2.1				
Giwa A	21	2.5	20	3.8		
Giwa B	3	0.1	10	0.6		
Halidawa	8	0.8	55	2.5		
Hubbare	1	4.6	3	1.1		
Madacci	1	1.7				
Magaryawa	24	1.3	24	2.8		
Magina	6	2.8	11	0.7		
Sabo Gari	0	0.0				
Sagi	3	1.4				
Sarkawa	20	3.2	11	1.5		
Sarkindoka A	52	2.3	55	1.1		
Sarkindoka B	15	2.1	4	0.6		
Sarkinruwa	12	3.3	1	0.1		
Sarkinwanki	2	0.8	7	2.8		
Shiyar Mai Gaza	1	2.1				
Shiyar Sage	9	3.7	25	2.0		
Wurno General Hospital	0	0.0				
Yardutse	6	2.3	6	0.4		

**Total**	**240**	**2.8**	**396**	**1.5**	**238**	

**Table 3.** Under 5 years population estimates, by settlement in the Magarya micro-census and Emergency Operations Center (EOC) house-to-house count.

Settlements	Micro-Census Count		EOC House-to-House Count	

	N	%	N	%
Unknown	25	1.6		
Akalawa	183	11.4	261	7.9
Alkanci	33	2.1		
Barkiya	19	1.2	187	11.3
Dafkawa	48	3.0	240	11.4
Dawarawa	15	1.0	153	17.5
Dutsin	52	3.3	85	11.8
Gida Garka	4	0.3		
Gida Kofar Giwa	18	1.1		
Giwa A	96	6.0	63	12.1
Giwa B	17	1.1	122	7.7
Halidawa	47	2.9	187	8.5
Hubbare	5	0.3	28	10.6
Madacci	7	0.4		
Magaryawa	203	12.7	60	6.9
Magina	43	2.7	171	10.6
Sabo Gari	7	0.4		
Sagi	19	1.2		
Sarkawa	107	6.7	81	11.1
Sarkindoka A	326	20.4	491	9.9
Sarkindoka B	138	8.6	77	11.2
Sarkinruwa	65	4.1	80	7.3
Sarkinwanki	14	0.9	70	28.0
Shiya Mai Gaza	10	0.6		
Shiya Sage	48	3.0	90	7.1
Wurno General Hospital	5	0.3		
Yardutse	45	2.8	100	6.6

**Total**	**1599**	**18.6**	**2546**	**9.7**

**Table 4.** Under-15 years population estimates, by settlement in the Magarya micro-census and Emergency Operations Center (EOC) house-to-house count.

Settlements	Micro-Census Count		EOC House-to-House Count	

	N	%	N	%
Unknown	66	1.6		
Akalawa	487	11.4	657	20.0
Alkanci	77	1.8		
Barkiya	64	1.5	331	20.0
Dafkawa	108	2.5	421	20.0
Dawarawa	35	0.8	175	20.0
Dutsin	113	2.7	144	20.0
Gida Garka	6	0.1		
Gida Kofar Giwa	50	1.2	255	20.0
Giwa A	284	6.7	104	20.0
Giwa B	59	1.4	317	20.0
Halidawa	119	2.8	441	20.0
Hubbare	8	0.2	53	20.0
Madacci	23	0.5		
Magaryawa	602	14.1	174	20.0
Magina	108	2.5	322	20.0
Sabo Gari	13	0.3		
Sagi	80	1.9		
Sarkawa	265	6.2	146	20.0
Sarkindoka A	791	18.5	990	20.0
Sarkindoka B	379	8.9	138	20.0
Sarkinruwa	188	4.4	220	20.0
Sarkinwanki	50	1.2	50	20.0
Shiyar Mai Gaza	21	0.5		
Shiyar Sage	128	3.0		
Wurno General Hospital	30	0.7		
Yardutse	117	2.7	303	20.0

**Total**	**4271**	**49.7**	**5241**	**20.0**

**Table 5.** Total population size of Magarya ward, by source of denominator data.

Micro-Census Count	GeoPoDe Estimate	2006 Census Projections	EOC House-to-House Estimate
8601	8708	16,994	26,205

**Appendix A.**
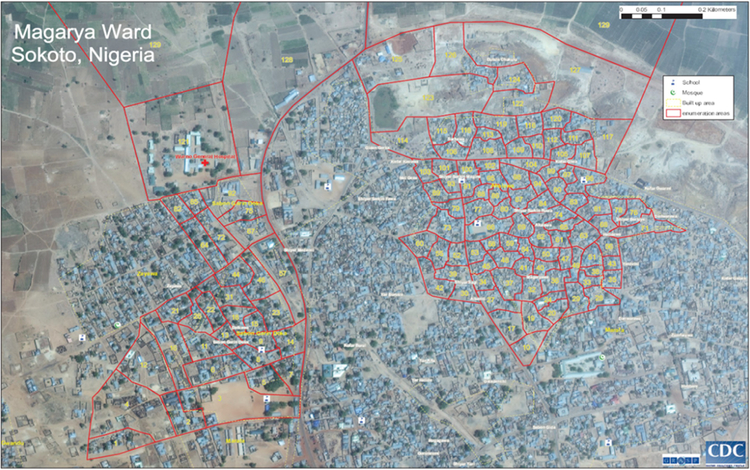
Satellite image of Magarya ward, divided into 129 enumeration areas.
